# Landscape of protein-protein interactions during hepatitis C virus assembly and release

**DOI:** 10.1128/spectrum.02562-22

**Published:** 2024-01-17

**Authors:** Alina Matthaei, Sebastian Joecks, Annika Frauenstein, Janina Bruening, Dorothea Bankwitz, Martina Friesland, Gisa Gerold, Gabrielle Vieyres, Lars Kaderali, Felix Meissner, Thomas Pietschmann

**Affiliations:** 1 Institute of Experimental Virology, TWINCORE, Centre for Experimental and Clinical Infection Research, Hannover, Lower Saxony, Germany; 2 RG Experimental Systems Immunology, Max-Planck Institute for Biochemistry, Planegg, Bavaria, Germany; 3 Department of Biochemistry & Research Center for Emerging Infections and Zoonoses (RIZ), University of Veterinary Medicine Hannover, Hannover, Lower Saxony, Germany; 4 Department of Clinical Microbiology, Virology, Umeå University, Umeå, Sweden; 5 Wallenberg Centre for Molecular Medicine (WCMM), Umeå University, Umeå, Sweden; 6 Junior Research Group “Cell Biology of RNA Viruses,” Leibniz Institute of Experimental Virology, Hamburg, Germany; 7 Institute of Bioinformatics, University Medicine Greifswald, Greifswald, Germany; 8 Systems Immunology and Proteomics, Institute of Innate Immunity, Medical Faculty, University of Bonn, Bonn, Germany; Indian Institute of Science, Bangalore, Karnataka, India

**Keywords:** hepatitis C virus, viral assembly and release, proteomics, affinity purification, endoplasmic reticulum, ERAD, HSPA5, Rad23B, host-pathogen interactions, virus-host interactions, lipoproteins

## Abstract

Assembly of infectious hepatitis C virus (HCV) particles requires multiple cellular proteins including for instance apolipoprotein E (ApoE). To describe these protein-protein interactions, we performed an affinity purification mass spectrometry screen of HCV-infected cells. We used functional viral constructs with epitope-tagged envelope protein 2 (E2), protein (p) 7, or nonstructural protein 4B (NS4B) as well as cells expressing a tagged variant of ApoE. We also evaluated assembly stage-dependent remodeling of protein complexes by using viral mutants carrying point mutations abrogating particle production at distinct steps of the HCV particle production cascade. Five ApoE binding proteins, 12 p7 binders, 7 primary E2 interactors, and 24 proteins interacting with NS4B were detected. Cell-derived PREB, STT3B, and SPCS2 as well as viral NS2 interacted with both p7 and E2. Only GTF3C3 interacted with E2 and NS4B, highlighting that HCV assembly and replication complexes exhibit largely distinct interactomes. An HCV core protein mutation, preventing core protein decoration of lipid droplets, profoundly altered the E2 interactome. In cells replicating this mutant, E2 interactions with HSPA5, STT3A/B, RAD23A/B, and ZNF860 were significantly enhanced, suggesting that E2 protein interactions partly depend on core protein functions. Bioinformatic and functional studies including STRING network analyses, RNA interference, and ectopic expression support a role of Rad23A and Rad23B in facilitating HCV infectious virus production. Both Rad23A and Rad23B are involved in the endoplasmic reticulum (ER)-associated protein degradation (ERAD). Collectively, our results provide a map of host proteins interacting with HCV assembly proteins, and they give evidence for the involvement of ER protein folding machineries and the ERAD pathway in the late stages of the HCV replication cycle.

**IMPORTANCE:**

Hepatitis C virus (HCV) establishes chronic infections in the majority of exposed individuals. This capacity likely depends on viral immune evasion strategies. One feature likely contributing to persistence is the formation of so-called lipo-viro particles. These peculiar virions consist of viral structural proteins and cellular lipids and lipoproteins, the latter of which aid in viral attachment and cell entry and likely antibody escape. To learn about how lipo-viro particles are coined, here, we provide a comprehensive overview of protein-protein interactions in virus-producing cells. We identify numerous novel and specific HCV E2, p7, and cellular apolipoprotein E-interacting proteins. Pathway analyses of these interactors show that proteins participating in processes such as endoplasmic reticulum (ER) protein folding, ER-associated protein degradation, and glycosylation are heavily engaged in virus production. Moreover, we find that the proteome of HCV replication sites is distinct from the assembly proteome, suggesting that transport process likely shuttles viral RNA to assembly sites.

## INTRODUCTION

Hepatitis C virus (HCV) has chronically infected more than 58 million individuals worldwide (1). Exposure to HCV usually leads to chronic, lifelong infections [the chronification rate is up to ca. 55%–85% (2)]. More than 10 years ago, the first directly targeting HCV antivirals became available, and today’s combination therapies cure almost 100% of treated individuals (3). Nevertheless, the incidence of HCV infection remains high with ca. 1.5 million new infections per year. An estimated 290,000 individuals died due to HCV-associated sequelae in 2019 emphasizing the severe disease burden caused by this virus (1). A prophylactic vaccine is not yet available, and its development is complicated due to the extreme diversity of HCV and viral immune evasion mechanisms.

The lipid-enveloped HCV particles comprise the viral core protein encasing the genomic RNA, viral envelope proteins E1 and E2, and human lipoproteins and lipids, giving them an unusually low and heterogeneous density (4). Understanding of the processes that shape these peculiar HCV “lipoviral” particles in the infected host cell is emerging (5, 6). They involve numerous viral and cellular factors, and they engage distinct subcellular compartments, including the endoplasmic reticulum (ER), cellular lipid droplets, and the secretory pathway. Viral factors essential for particle production accumulate on ER membranes. This includes the E1-E2 proteins, which, once incorporated into virions, mediate cell entry and receptor interactions (7, 8). HCV core also originates at the ER; however, it traffics from there to cellular lipid droplets (LDs) (9, 10). The p7 protein, a viroporin important for an HCV assembly step prior to capsid formation and membrane envelopment (11–13), accumulates at the ER (14, 15). E1-E2 and p7 proteins are known to interact with NS2 (16), which is dispensable for RNA replication but essential for infectious virus production (17, 18). The remaining nonstructural proteins NS3, 4A, 4B, NS5A, and NS5B establish membrane-bound replication complexes catalyzing genome replication (19).

Besides these viral factors, cellular factors, particularly those involved in lipoprotein synthesis, also participate in HCV assembly (20). Studies have implicated proteins involved in production of very-low-density lipoprotein in HCV particle production. These factors include apolipoprotein B, apolipoprotein E (ApoE), apolipoprotein C1, and microsomal triglyceride transfer protein (21–26). Moreover, diacylglycerol acyltransferase-1, a cellular enzyme needed for fueling lipid droplets with neutral lipids, is necessary for transport of core to LDs and for production of infectious viral progeny (27). The importance of LDs in HCV virion production is further supported by the observation that alpha-beta hydrolase domain containing 5 and patatin-like phospholipase domain containing 2 (also known as ATGL), which act in concert to mobilize lipids from lipid droplets, act as cellular assembly co-factors (28, 29).

Collectively, this previous work highlights complex and dynamic cellular processes involving several cellular compartments and membrane microdomains, which participate in production of infectious HCV particles. How protein interactions of viral and host proteins are regulated to facilitate HCV particle production is incompletely understood. Mass spectrometry-based proteomics provide a powerful tool to dissect the molecular mechanisms governing assembly of proteins in viral infections (30–32). Here, we aimed to describe the composition of protein complexes interacting with essential viral and host factors driving HCV particle production. Additionally, we used viral mutants, carrying mutations disrupting HCV assembly at distinct stages, to understand how protein complexes may change in the course of assembly. Taken together, this high-resolution proteomic and functional analysis reveals numerous novel HCV-host protein interactions and implicates new host pathways involved in assembly of virions.

## RESULTS

### Viral constructs used for the analysis of the HCV assembly proteome

**Fig 1:**
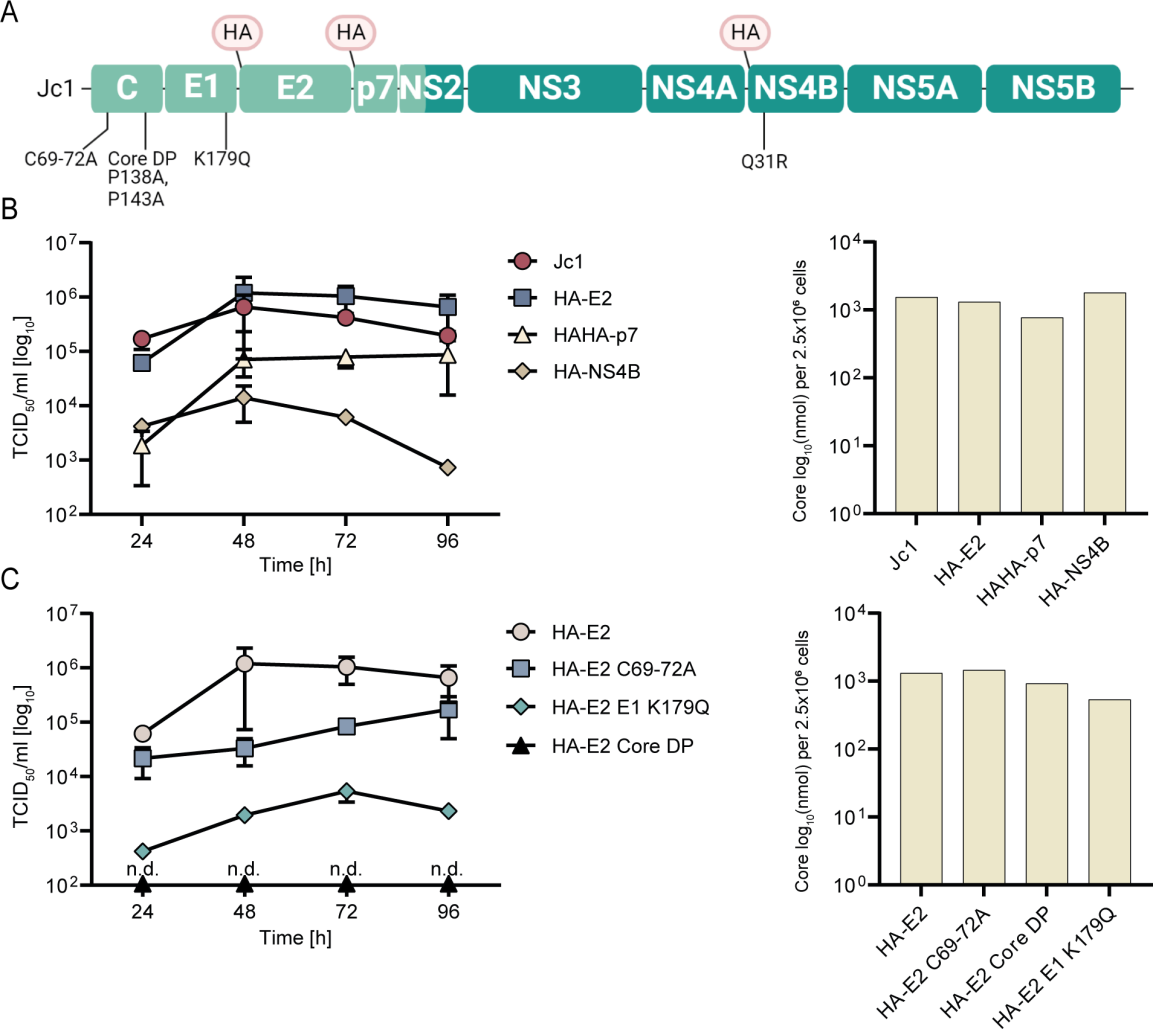
Properties of viral constructs used for analysis of the HCV assembly proteome. (**A**) Schematic overview of the HCV genome. HA- or double HA-tag insertion sites are depicted above the viral polyprotein. Mutations introduced in specific constructs are indicated below. (**B**) Influence of HA-tag insertion on infectious virus production. We quantified infectious virus production using a limiting dilution assay. Mean values of three independent biological replicates and the standard deviation are given. We transfected given HCV constructs into Huh-7.5 cells and monitored transfection efficiency by a core-specific enzyme-linked immunosorbent assay (ELISA) (*n* = 1; at 48 hours, right panels in **B** and **C**). (**C**) Influence of given point mutations on infectious virus production of the HA-E2 virus construct. Experimental setup as in panel **B**. (**B and C**) Mean ± SD.

To obtain a comprehensive overview of proteins potentially involved in HCV particle production, we analyzed the protein-protein interactions of both viral and host proteins critical for assembly. We focused on HCV E2, p7, and cellular ApoE because they represent different functional players in virus production, including proteins that are abundant components of mature particles (E2 and ApoE). We also analyzed the NS4B protein interactome to distinguish the assembly-devoted protein complexes from those formed around NS4B, an integral component of ER-derived double membrane vesicles engaged in RNA replication. Since E2 re-localizes during HCV assembly (33), we also assessed its proteome in cells replicating viral mutants, which arrest HCV assembly by different mechanisms, and, therefore, could inform about changes in the E2 interaction network during particle production. First, we used a viral mutant carrying a P138A, P143A double mutation within the core protein (core DP) (34–36) (Fig. 1A). This mutation prevents complete trafficking of core protein to LDs, ablating infectious virus production at a very early stage. Second, we employed a virus with four consecutive amino acids within core changed to alanines (core C69-72A) (37). This mutation causes accumulation of HCV core protein on the surface of LDs, reducing infectious virus production by more than 90%, likely by impairing capsid recruitment into nascent viral particles and particle membrane envelopment (13). Third, we analyzed the proteome of E2 in cells replicating a virus with a mutation in the transmembrane domain of E1 (E1 K179Q). This mutation impairs E1-E2 heterodimerization (38). Although this alteration does not much affect production and release of virus particles, secreted virions are less infectious (11, 13).

To ensure comparable experimental conditions, we used the hemagglutinin tag (HA-tag) as a common epitope tag in all our affinity purifications. Figure 1A schematically depicts all virus constructs used in this study. Figure 1B and C summarize infectious virus production obtained after transfection of these constructs into Huh-7.5 cells. As was reported previously, insertion of the HA-tag at the N-terminus of E2 was well tolerated and did not attenuate infectious virus production, whereas tagging of p7 (HAHA-tag) and NS4B diminished infectious progeny (Fig. 1B) (39, 40). We used a virus variant with a coding Q31R mutation in HA-NS4B that partially recovers the RNA replication defect of the epitope-tagged viral mutant. In agreement with previous studies, the core DP mutation completely ablated infectious virus production, whereas the core C69-72A mutant produced less than 10% viral progeny and infectious titers of the E1 K179Q mutant declined more than 100-fold compared to those of the parental Jc1-HA-E2 virus (Fig. 1C) (11, 13, 35). These defects were not due to different transfection, protein translation, or RNA replication, since intracellular core protein accumulated to comparable levels (Fig. 1C, right panel). To analyze the ApoE protein interactome in HCV-producing cells, we used a Huh-7.5-derived cell line with shRNA knock-down of endogenous ApoE and restored virus production by expression of HA-ApoE (41).

### HCV assembly interactomes

**Fig 2:**
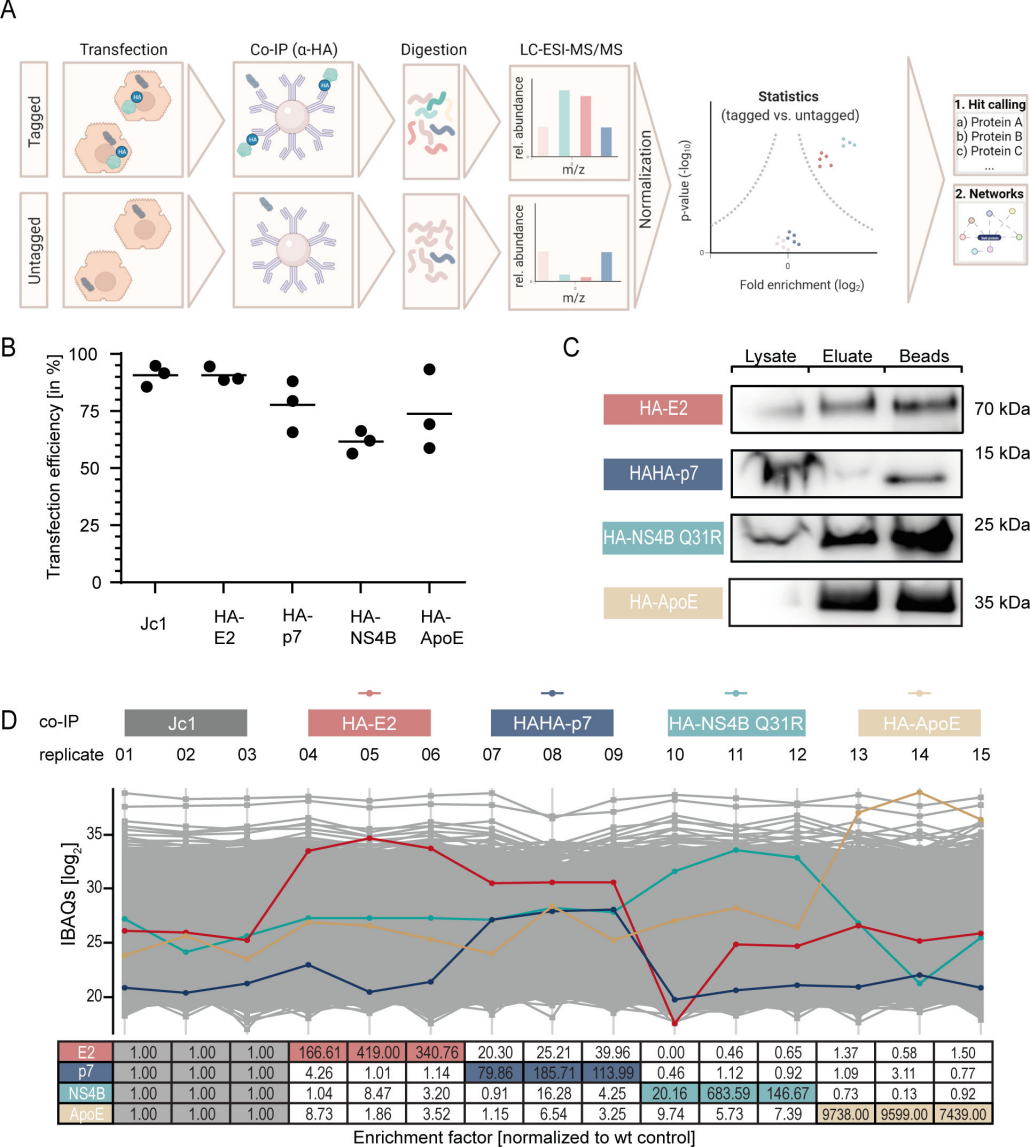
Mass spectrometry workflow and quality control. (**A**) Scheme of the workflow of our lLiquid chromatography electrospray ionization tandem mass spectrometry (LC-ESI-MS/MS) proteomics approach. (**B**) We controlled the transfection efficiency of Huh-7.5 cells 24 hours post transfection (hpt) with viral RNA generated via *in vitro* transcription (IVT), by using indirect immunofluorescence staining with an anti-NS5A antibody. The percentage of NS5A-positive cells is given for each biological replicate. The horizontal line represents the mean value (*n* = 3). (**C**) Huh-7.5 cells transfected with the indicated constructs were lysed 48 hpt. We confirmed the presence of the given prey proteins in the total lysate, the eluate, and on the beads after elution by immunoblotting. (**D**) We determined the abundance of proteins by mass spectrometry using the MaxQuant software to calculate intensity-based absolute quantification (IBAQ) values as a measure of protein abundance [IBAQ (log_2_)] (42, 43). Total protein amounts of HA-E2 (red), HAHA-p7 (dark blue), HA-NS4B Q31A (light blue), and HA-ApoE (beige) are given for all IPs. Mean enrichment factors of these proteins were calculated by comparing protein abundance in immune precipitations from epitope-tagged samples to untagged control samples (*n* = 3).

**Fig 3:**
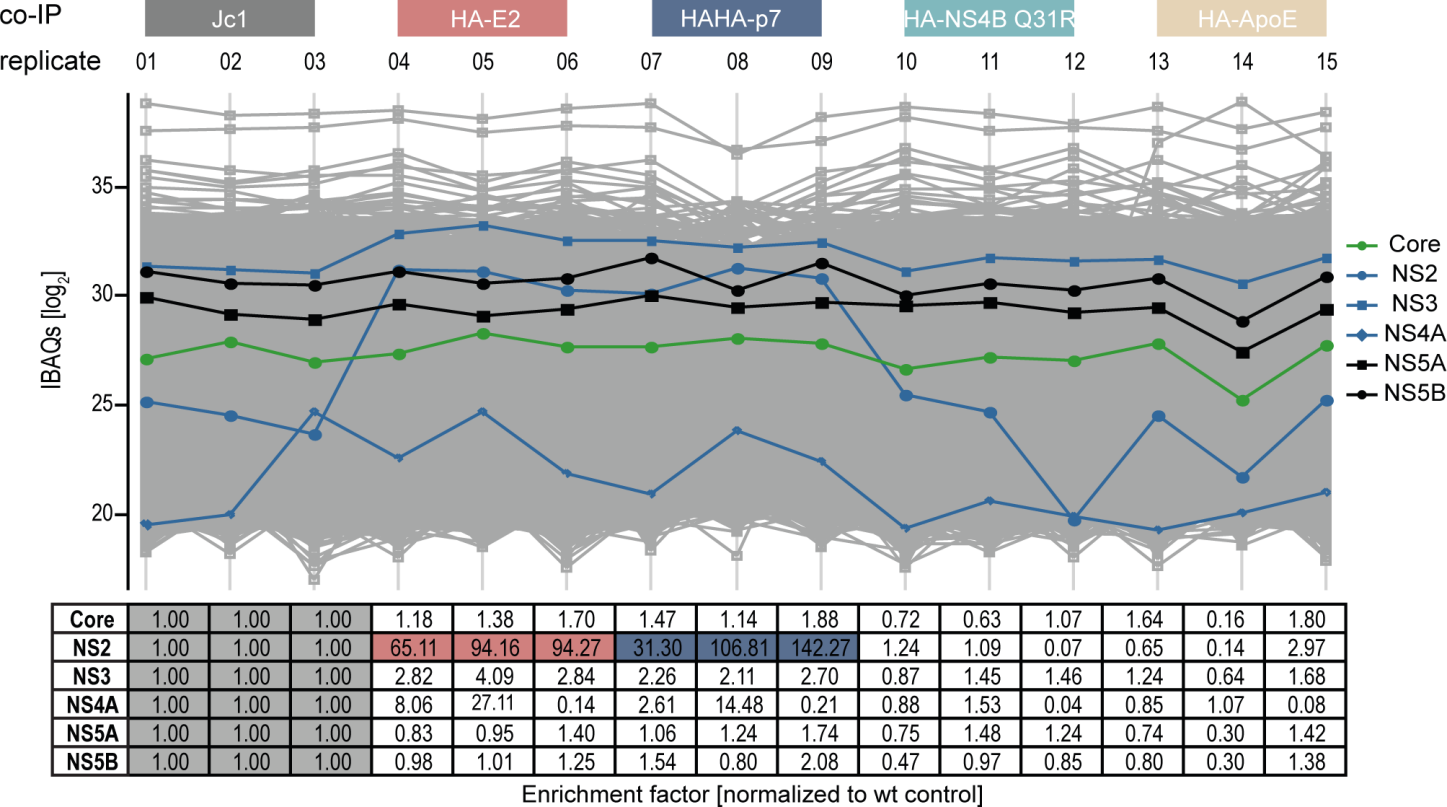
Profile plot of quantified HCV proteins in co-IPs performed with anti-HA antibody-tagged beads. Total protein abundance is depicted for each viral protein (legend) for all performed co-IPs. In the table below, the fold change of protein abundance between the respective bait proteins and the corresponding untagged Jc1 control is shown. Boxes containing values of controls for viral proteins are highlighted in light gray. Red boxes indicate the enrichment of NS2 with E2 and blue with p7 (*n* = 3).

We used label-free mass spectrometry for identification and quantification of HA-E2, HAHA-p7, HA-ApoE, and HA-NS4B protein interactomes (Fig. 2A). Therefore, we transfected Huh-7.5 cells with the tagged viral construct (e.g., Jc1-HA-E2) or the untagged control virus Jc1. In the case of HA-ApoE proteome analysis, we transfected parental Jc1 into either Huh-7.5/HA-ApoE cells (41) or parental Huh-7.5 cells. This approach ensured identification of specific binders based on enriched peptide signals in the tagged replicates relative to the ones conducted on the untagged control samples. Forty-eight hours after transfection, we prepared cellular extracts and affinity-purified protein complexes. For all three biological replicates, we controlled transfection efficiency by immunofluorescence staining to ensure similar transfection levels (Fig. 2B). We also controlled the purification procedure by immunoblots against the respective HA-tagged bait proteins (Fig. 2C) and determined the composition of purified protein complexes. The enrichment efficiency of each of our four bait proteins across all three biological replicates and relative to the untagged control baits is summarized by the respective intensity-based absolute quantification (IBAQs) in Fig. 2D. IBAQ values indicate the total amount of measured bait proteins in the mass spectrometry runs. These data confirmed a strong enrichment of our bait proteins: in the precipitation of HAHA-p7, we noted a ca. 20- to 40-fold co-enrichment of E2 protein and possible precursors indicative of the interaction between these two viral proteins (Fig. 2D). Vice versa, we did not find a co-enrichment of p7 in the HA-E2 precipitation. The reason for this is not known. However, we recently noted that the HA-tag at the N-terminus of p7 decreases processing at the p7-NS2 site (39). Although we did not investigate this, it is possible that the HA-tag also reduces processing at the E2-p7 site, which may lead to enhanced co-precipitation of E2 with the HAHA-tagged p7. Finally, NS2 co-precipitated with both HA-E2 and HAHA-p7, which is visible in the profile plot depicting enrichment of HCV proteins in HA-tag co-immunoprecipitations (co-IPs) (Fig. 3).

**Fig 4:**
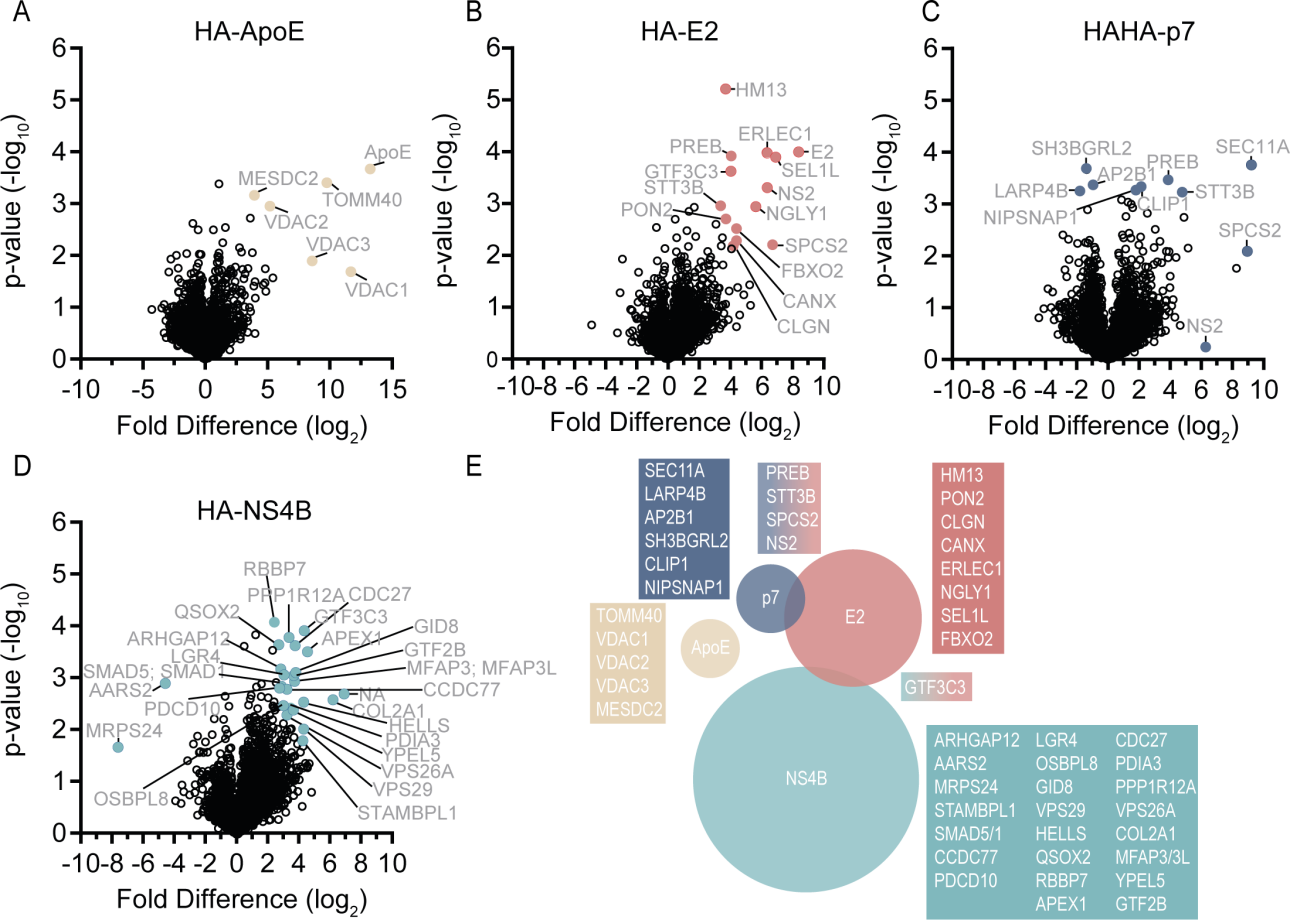
Interactomes of cellular ApoE and viral E2, p7, and NS4B proteins in liver cells producing infectious HCV. Volcano plots of interacting proteins of HA-tagged bait proteins ApoE (**A**), E2 (**B**), p7 (**C**), and NS4B Q31R (**D**), which were compared to untagged Jc1 for analyses (**B–D**) or Huh-7.5 cells with an endogenous ApoE expression (**A**). (**A–D**) We applied a two-sided *t*-test for statistical analysis. Significant interactors (Welch’s *t*-test; FDR ≤0.1, S0 = 1, results from three independent biological replicates). (**E**) Venn diagram of host factors interacting with bait proteins E2 (red), p7 (dark blue), NS4B (light blue), and ApoE (beige). Factors specific for one interactor or interacting with more than one bait protein are depicted.

Next, we quantified the co-enrichment of proteins from tag-containing lysates versus lysates infected with untagged virus. Thereby, we could specifically identify proteins in complex with our tagged bait proteins. For the identification of bait protein interactors, we performed standard statistical testing (two-sided *t*-test) at a stringent false discovery rate (FDR) to correct for multiple hypothesis testing (44). The volcano plots displayed in Fig. 4A through D highlight proteins co-precipitating with our bait proteins HA-E2, HAHA-p7, HA-NS4B, and HA-ApoE, respectively. Significant interactors are depicted in a Venn diagram in Fig. 4E. Factors enriched with more than one bait protein are highlighted in dual-colored boxes.

### Primary E2 interactome and changes of the E2 interactome caused by core protein mutations

**Fig 5:**
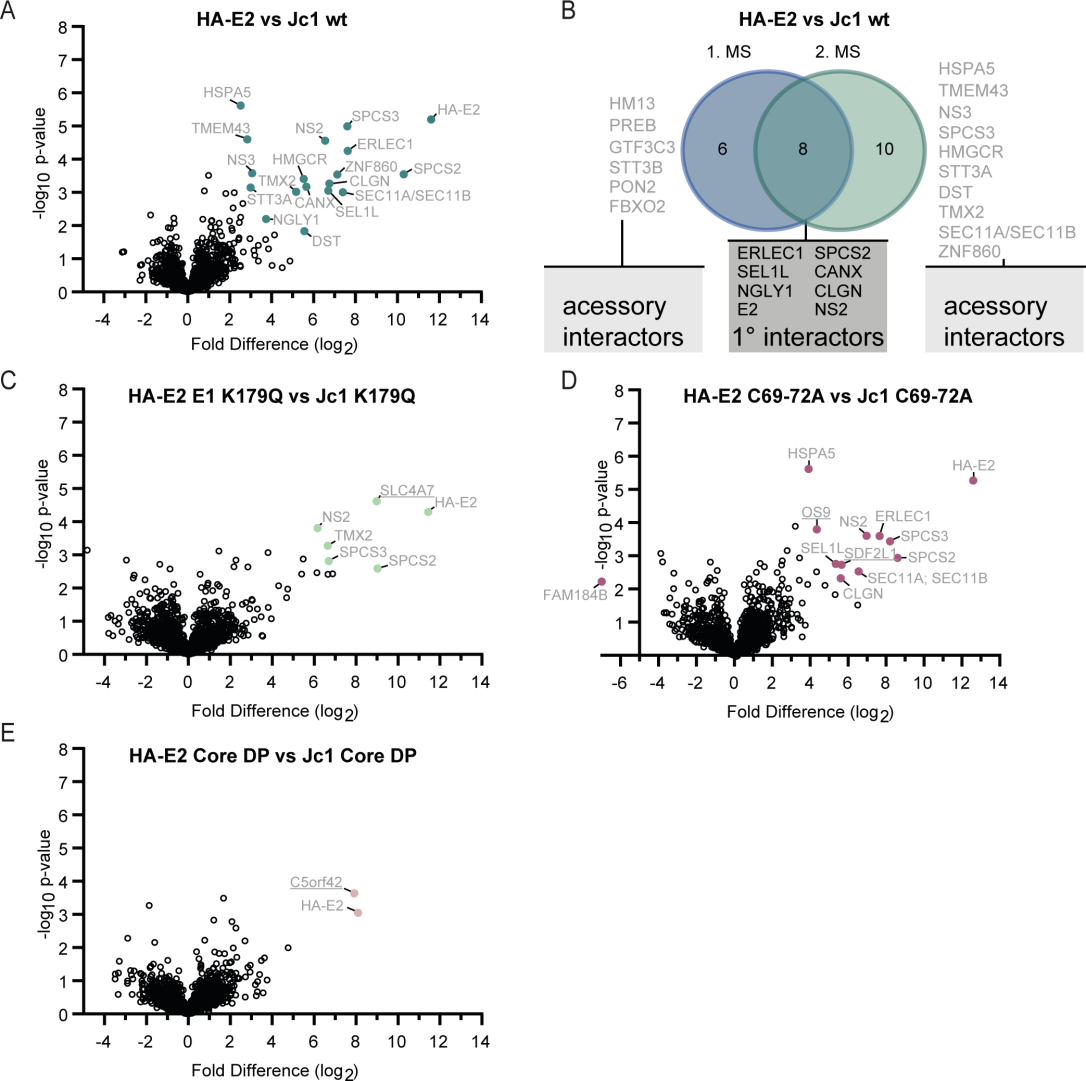
The HCV E2 primary interactome and changes to E2 protein interactions caused by assembly disrupting mutations. (**A**) Volcano plot of HA-E2 wt versus Jc1 wt virus. (**B**) Venn diagram comparing the HA-E2 interactomes from the first and second analyses. We defined those factors significantly binding in both screens as the primary E2 interactome and the ones we found in at least one of the screens as accessory interactors. (**C–E**) Volcano plots of mutant virus E2 interactomes. We identified E2 binding proteins of the indicated HA-E2 tagged virus mutants and used the cognate untagged viruses as control. (**A and C–E**) A two-sided *t*-test was applied. (FDR ≤0.05; S0 = 1; three valid values in the first group, *n* = 3–4). (**C–E**) Novel HA-E2 binders are underlined.

Subsequently, we used the same workflow to investigate HA-E2 protein interactions of viral mutants arrested at distinct stages of HCV particle assembly (Fig. 1 and 5). To identify specific binders, we compared each HA-E2-tagged viral mutant with its corresponding control virus lacking the HA-epitope tag at the E2 protein. In parallel, we repeated the HA-E2 interaction analysis of the parental Jc1 HA-E2 wild-type virus, as a control, to directly compare the mutation-dependent changes to the HA-E2 proteome recorded in the same workflow. The HA-specific antibodies co-precipitated 18 proteins from cells replicating the parental Jc1-HA-E2 virus (Fig. 5A and B). These included viral HA-E2, NS2, and NS3 and 15 host proteins, six of which we had also discovered in the first proteome analysis of the HA-E2 interactome (compare Fig. 4B and E, and Fig. 5A and B). We defined these six E2-host protein interactors plus NS2, which we confirmed in a total of seven independent biological replicates, as the HCV-E2 primary interactome (Fig. 5B, 1° interactors). This second mass spectrometry analysis of the HA-E2 interactome identified nine additional host proteins and NS3, which were not among the significant interactors of the first HA-E2 analysis (compare Fig. 5A through E with Fig. 4B and E). Vice versa, the first analysis of the HA-E2 interactome had implicated six E2 binders, which were not among the significant E2 interactors in the second analysis. We defined these 16 additional proteins as the HCV-E2 accessory interactors (15 host proteins and the viral NS3 protein). Note that in our MS peptide analysis, we were unable to distinguish between signal peptidase complex catalytic subunits SEC11A and SEC11B, so either one of the proteins or both interacted with HA-E2.

**Fig 6:**
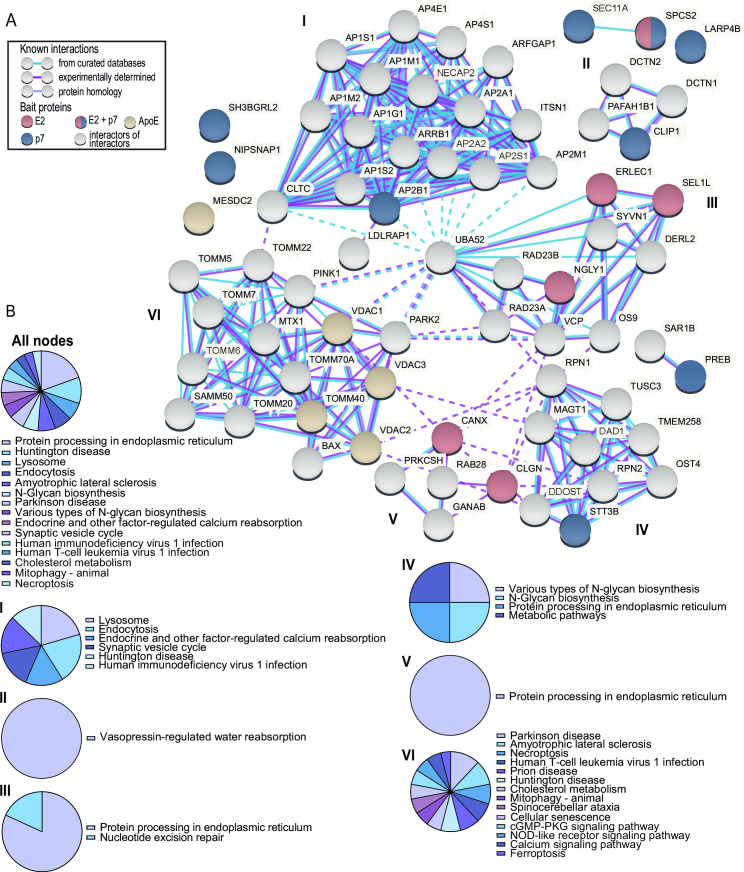
STRING and KEGG pathway analyses of the HCV assembly proteome. (**A**) STRING interaction network of host factors binding to HA-E2 (only primary HA-E2 interactors were considered), HAHA-p7, and HA-ApoE (confidence of 0.4 and no more than 50 interactors). Nodes are color coded based on the interaction partners co-precipitating the respective host protein: E2 (red), p7 (blue), and ApoE (yellow). Markov cluster algorithm clustering was performed (inflation parameter = 3). Lines indicate defined clusters, which were then subjected to pathway analysis. Dotted lines indicate connection between the different clusters. (**B**) KEGG annotated pathways, which are enriched for the factors occurring in this STRING network, including all depicted nodes (B I) for upper left cluster I, (B II) for the upper right cluster, (B III) for the middle right cluster III, (B IV) for the bottom right cluster IV, (B V) for the bottom middle cluster V, and (B VI) for the middle left cluster VI.

To define host protein interaction networks and functional cellular protein machineries coordinating HCV assembly and release, we conducted a STRING network analysis. For this network, we used the interactors of HCV assembly co-factors HA-ApoE, HAHA-p7, and HA-E2 (primary interactors) as input (Fig. 6). The host proteins interacting with binding partners of HA-E2, HAHA-p7, and HA-ApoE (colored nodes in Fig. 6A) clustered in six distinct interaction networks (I–VI) representing a range of Kyoto Encyclopedia of Genes and Genomes (KEGG) functional pathways (Fig. 6B). Networks III, IV, and V comprise five of the six primary HA-E2 interactors. The dominant functional pathways characterizing these networks are protein processing in the endoplasmic reticulum and N-glycan biosynthesis. Cluster II also contained nucleotide excision repair as a functional pathway, since RAD23A and B are present in this network. Both proteins contain ubiquitin-like domains and regulate proteasome assemblies (45). RAD23B interacts with proteasomes, contains two ubiquitin binding motifs, and serves as a shuttle of tagged proteins to the proteasome (45, 46). Notably, Kim et al. reported that the yeast orthologue of RAD23B is involved in the ER-associated protein degradation (ERAD) pathway. It regulates glycoprotein turnover through interactions with regulators of proteolysis by transfer of ubiquitinated substrates to the proteasome (47, 48).

The sole primary HA-E2 interactor, which also bound to HAHA-p7, was SPCS2. It mapped outside of the six interaction networks and is known to bind SEC11 homolog A, the signal peptidase complex subunit SEC11A, which is also an HAHA-p7 binding protein (Fig. 6B). Additional HAHA-p7 interactors mapped to cluster IV (STT3 oligosaccharyltransferase complex catalytic subunit B; STT3B), cluster I (adaptor related protein complex 2 subunit beta 1; AP2B1), and cluster II (CAP-Gly domain containing linker protein 1; CLIP1).

We noted that each of the virus assembly-disrupting mutations had a profound impact on the HA-E2 interactome. In general, all viral mutants exhibited an HA-E2 interactome with reduced diversity compared to the parental virus: we found only 5, 10, and 1 significant binding partner(s) for the HA-E2 E1 K179Q, the HA-E2 C69-72A, and the HA-E2 core DP mutants, respectively (Fig. 5C through E). These prominent changes included loss of interactions with members of the primary and accessory HCV HA-E2 interactome as well as the gain of novel interactors such as sodium bicarbonate cotransporter 3 SLC4A7 for the HA-E2 E1 K179Q mutant, osteosarcoma amplified 9 (OS9) and SDF2L1 for the HA-E2 C69-72A mutant, and C5orf42 in case of HA-E2 core DP. These changes were most prominent for core DP mutant, arresting assembly at an early stage, and least extensive for the HA-E2 E1 K179Q mutant.

**Fig 7:**
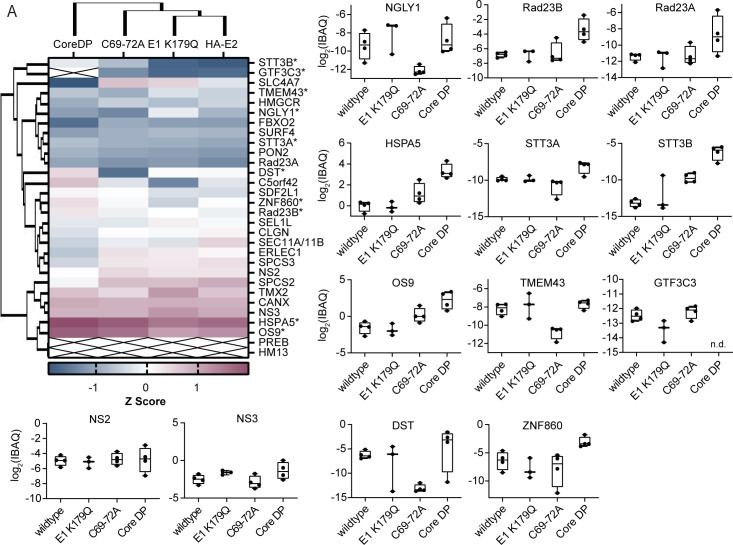
HCV core and E1 mutations modulate the HA-E2 protein interactome. Heat map showing alterations of the E2 interactome between the mutant viruses. All primary and accessory HA-E2 binders and RAD23A and B were plotted. For statistical testing, we used multi-parametric two-way analysis of variance (ANOVA) (FDR ≤0.05; S0 = 0; second ANOVA *P*-value ≤0.05). Significant interactomic changes are highlighted with asterisks. Boxplots on the right show IBAQ values of those proteins whose HA-E2 interaction significantly changed between the viral mutants. Values were normalized to wt E2 (bait) (*n* = 3–4, mean ± SD).

To visualize these changes, we next plotted the Z-scores derived from IBAQ values from all significant HA-E2 interactors across all four different viral constructs into a clustered heat map showing virus mutant-dependent changes of the HA-E2 interactome (Fig. 7A). Speculating that some of these mutations may affect HCV E1-E2 protein folding, transport, and glycosylation, we also included Rad23A and B, members of the interaction network cluster II and host factors involved in ERAD and protein shuttling to the proteasome, into this analysis (Fig. 6A). The heat map revealed significant, mutant-specific changes for 10 HA-E2 interactors. In general, most prominent changes occurred for the core DP mutant: this mutation significantly enhanced six HA-E2 interactions including Rad23B, Rad23A, HSPA5, STT3A, STT3B, OS9 (ERLEC2), and ZNF860. Most of these proteins are part of the STRING interaction cluster III and adjacent clusters (Fig. 6). In contrast, HA-E2 protein interactions with peptide-N(4)-(N-acetyl-beta-glucosaminyl)asparagine amidase (NGLY1), TMEM43, and DST decreased for the C69-72A mutant. NGLY1 is one of the primary HA-E2 interactors, detected in both screens, which also maps to the KEGG cluster II. With the exception of ZNF860, the mRNAs of these HA-E2 interactors are well expressed in primary human hepatocytes [Table 1, comprising data published in reference (49)]. These results suggest that altering distinct core protein features, which influence core protein trafficking to and from lipid droplets, significantly modulates the HA-E2 interactome.

**TABLE 1:** Primary human hepatocyte (PHH) RNAseq expression data of interaction candidates adapted from reference (49)^*a*^

Factors	MS screen	Interactome	Transcriptome (mean RPKM)
HM13	1	HA-E1 wt	9,700067186
ERLEC1	1 + 2	HA-E1 wt, HA-E2 C69-72A	26,71941095
PREB	1	HA-E1 wt	11,17734533
GTF3C3	1	HA-E1 wt	3,19956537
STT3B	1	HA-E1 wt	30,49051233
PON2	1	HA-E1 wt	18,69401667
SEL1L	1 + 2	HA-E1 wt, HA-E2 C69-72A	39,31439162
NGLY1	1 + 2	HA-E1 wt	4,345098734
SPCS2	1 + 2	HA-E1 wt, HA-E2 E1 K179Q, HA-E2 C69-72A	34,69252051
FBXO2	1	HA-E1 wt	N.D.
CANX	1 + 2	HA-E1 wt	191,350376
CLGN	1 + 2	HA-E1 wt, HA-E2 C69-72A	6,92743053
HSPA5	2	HA-E1 wt, HA-E2 C69-72A	884,5991617
TMEM43	2	HA-E1 wt	12,75660663
STT3A	2	HA-E1 wt	17,00506236
TMX2	2	HA-E1 wt, HA-E2 E1 K179Q	20,56274902
DST	2	HA-E1 wt	18,6126971
HMGCR	2	HA-E1 wt	15,11854724
SEC11A	2	HA-E1 wt, HA-E2 C69-72A	29,77906237
SEC11B	2	HA-E1 wt, HA-E2 C69-72A	N.D.
ZNF860	2	HA-E1 wt	N.D.
SPCS3	2	HA-E1 wt, HA-E2 E1 K179Q, HA-E2 C69-72A	12,00998976
SLC4A7	2	HA-E2 E1 K179Q	3,871523314
SDF2L1	2	HA-E2 C69-72A	16,33716977
OS9	2	HA-E2 C69-72A	45,62013374
C5orf42	2	HA-E2 core DP	RPKM <1
Rad23B	Interactor of NGLY1		50,70218963
Rad23A	Interactor of NGLY1		19,8987782
ApoE	Control		265,8809538
SCARB1	Control		8,141994532
LDLR	Control		15,70641848
CD81	Control		11,06836968
Albumin	Control		5672,707236

^*a*^ RPKM: reads per kilobase of transcript per million mapped reads, MS: mass spectrometry, N.D. not detected.

### Components of the HCV assembly proteome modulate HCV replication and assembly

**Fig 8:**
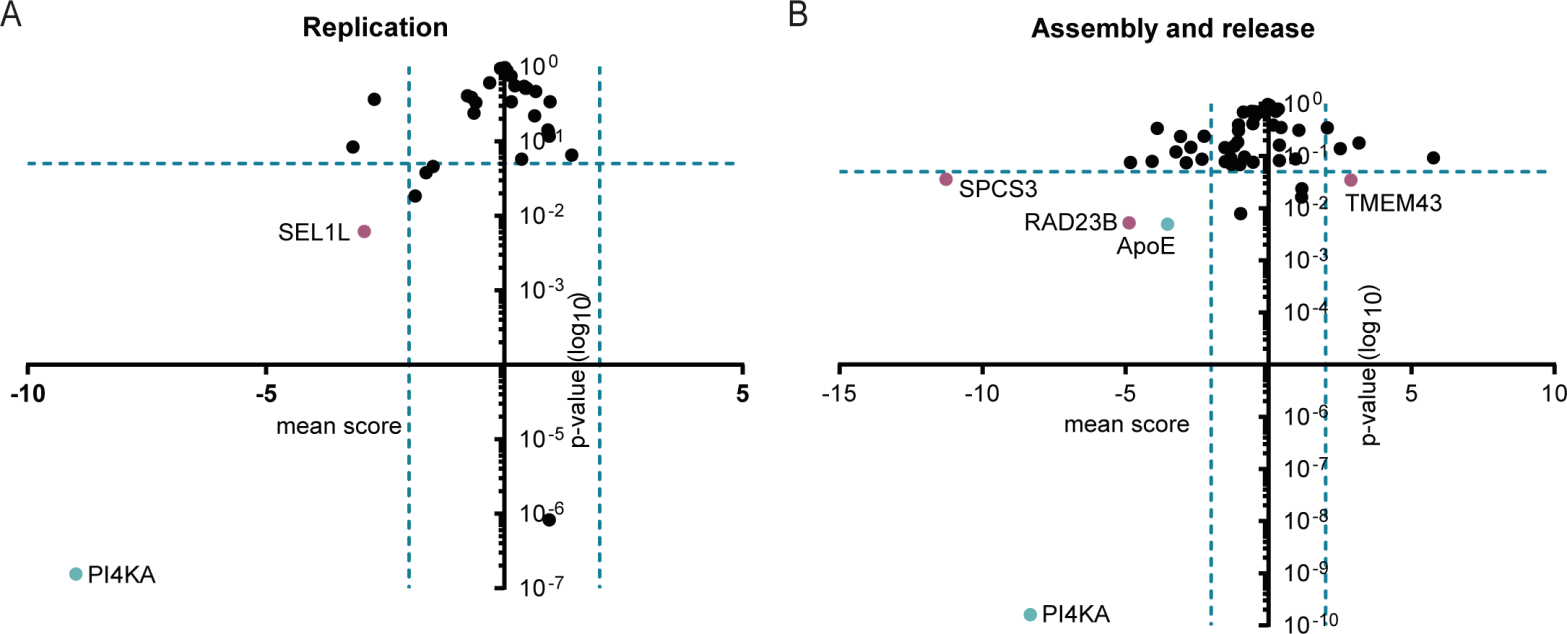
RNA interference screening of host factors of the HCV assembly proteome. Host factors identified as HA-E2 primary and accessory interaction partners (Fig. 5B), HAHA-p7, and HA-ApoE binders (Fig. 4) were silenced by RNA interference with a pool of three siRNAs per target. Moreover, SLC4A7, OS9, SDF2L1, and C5orf42, *de novo* HA-E2 interactors identified with at least one of the mutants, as well as Rad23A and RAD23B were included as interactors of NGLY1, an HA-E2 interactor. Subsequently, these cells were infected with the reporter virus JcR2a, and RNA replication was measured 48 hours later. The z-score of a knock-down was calculated by subtracting the median of the negative controls on the same plate and dividing by the standard deviations of the controls. Mean z-scores were calculated by averaging over knock-downs targeting the same gene. Values *x* < 0 indicate a reduction, and *x* > 0, an increase of HCV replication or assembly and release compared to the mean of controls. On the y-axis, the log_10_ of the *P-*values of the scores are plotted. (**A**) Infectious progeny produced at this time point were passed on to naïve cells, and infection efficiency reflecting assembly of new viral progeny in the silenced cells was quantified 48 hours post second round infection. (**B**) PI4KA and ApoE (green dots) were silenced as controls for host factors influencing RNA replication and assembly, respectively. Hit calling was based on mean score ≥2 and *P*-value ≥0.05; *t*-test was applied following the RNAither pipeline (50), *n* = 4–6. Host factors meeting these inclusion criteria are highlighted as red circles.

To test if members of the HCV assembly proteome participate in HCV life cycle steps, we silenced the mRNA expression of all the HA-E2 (primary and accessory interactors), HAHA-p7, and HA-ApoE interactors by RNA interference. We also included RAD23A and RAD23B into the screen because the STRING network showed their direct interactions with NGLY1 (Fig. 6A), an HA-E2 binding protein, and because the core DP mutation significantly enhanced Rad23B co-precipitation with HA-E2 (Fig. 7). As controls, we silenced expression of phosphatidylinositol 4-kinase alpha (PI4KA) and ApoE, host factors involved in RNA replication and assembly, respectively. We infected siRNA-treated Huh-7.5/F-Luc cells with JcR2a reporter virus and assessed effects on cell viability, HCV cell entry, and RNA replication 48 hours later using a dual luciferase assay. Moreover, we passed the culture fluid of these cells to naïve Huh-7.5/FLuc cells to quantify infectious virus production [Fig. 8; Materials and Methods (28, 29)]. Silencing of SEL1L and PI4KA significantly reduced HCV RNA replication (Fig. 8A), whereas silencing of SPCS3, RAD23B, ApoE, and PI4KA decreased and silencing of transmembrane protein 43 (TMEM43) slightly enhanced infectious virus production (Fig. 8B).

**Fig 9:**
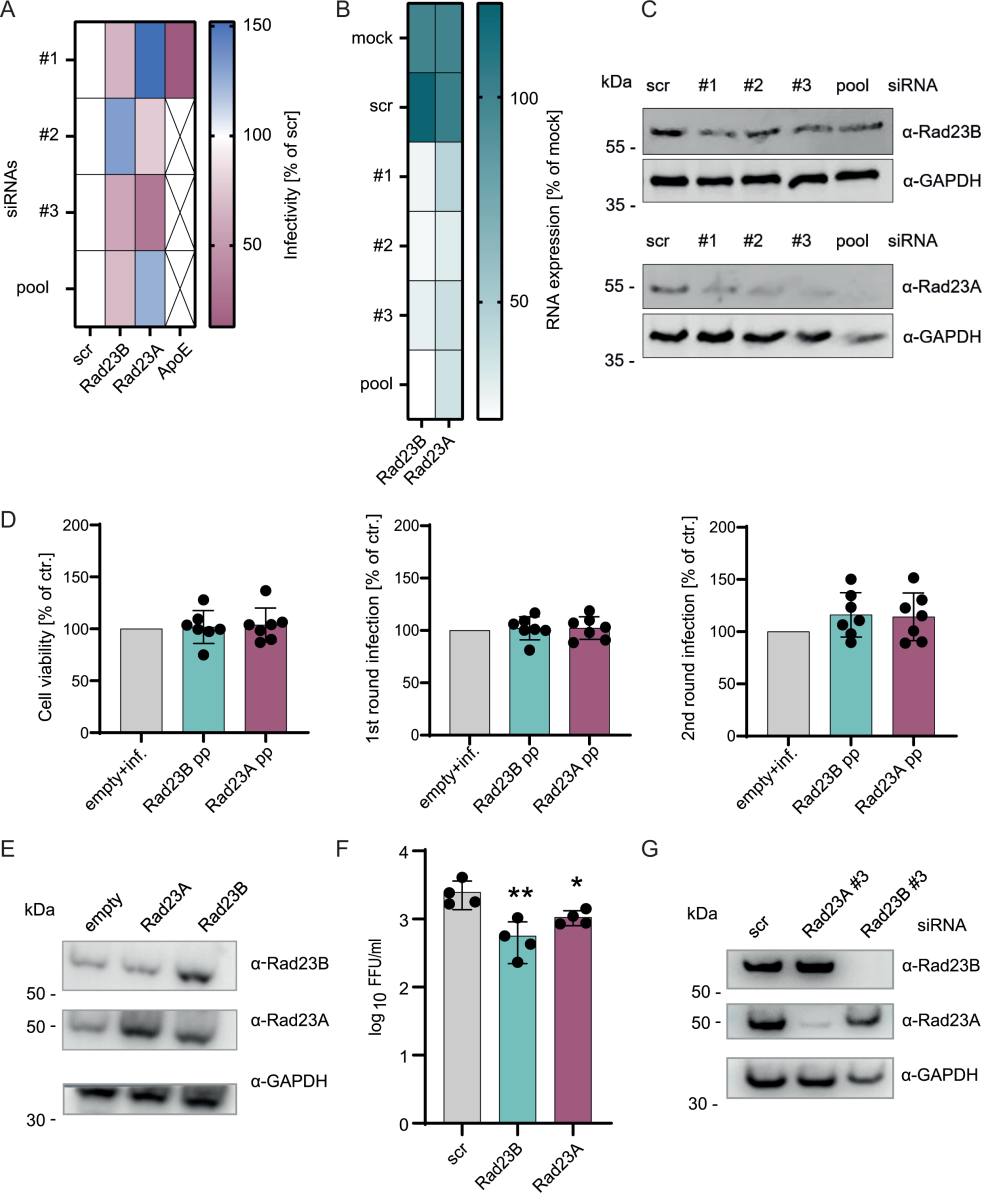
Modulation of RAD23A and B expression influences HCV infectious virus production. (**A**) We transfected Huh-7.5/FLuc cells with one of three different siRNAs per factor or a pool of these siRNAs. Forty-eight hours post transfection (hpt), we infected these cells with the reporter virus JcR2a. We passed the culture fluid of these cells to naïve Huh-7.5/Fluc cells and quantified infection 48 hours later. The heat map shows infection data normalized for cell viability and RNA replication quantified in the dual luciferase assay (*n* = 3). (**B**) In uninfected Huh-7.5 cells, we quantified the abundance of RAD23A and RAD23B mRNA by qRT-PCR and (**C**) Rad23A and Rad23B protein levels by Western blot at 48 hours post siRNA transfection. Western blot from one representative experiment is shown. (**D**) Huh-7.5/FLuc cells were transduced with an empty lentiviral vector or vectors encoding RAD23A or RAD23B. Forty-eight hpt, we infected the cells with JcR2a, and 48 hours later, we measured cell viability as well as cell entry/RNA replication. Culture supernatant was transferred to naïve Huh-7.5/FLuc cells to measure infectious virus production 48 hours post second round infection. Samples were normalized to infected cells treated with an empty control vector (*n* = 7). (**E**) Protein expression was analyzed 48 hpt (representative experiment). (**F and G**) Focus-forming unit (FFU) assay using the untagged HCV Jc1 strain in Huh7.5 cells. Cells were transfected with siRNA, and after 24 hours, cells were infected with a multiplicity of infection of 0.1 TCID_50_/cell . (**G**) Forty-eight hours post infection, cells were lysed, and Western blot was performed (representative experiment). (**F**) Supernatant of infected cells was titrated on naϊve Huh7.5 cells, and infections were stopped 48 hours later. Virus was fluorescently stained with an anti-NS5A antibody, FFUs were counted manually, and data were normalized to the scr control (*n* = 4) (mean ± SD, Kruskal-Wallis test, only significant differences are highlighted by an asterisk, *P < 0.05, **P < 0.005).

The importance of components of the signal peptidase complex such as SPCS1 and 3 for flavivirus particle production and HCV was previously recognized and described (51, 52). Zhang and colleagues reported protein sel-1 homolog 1 (SEL1L) as host factor important for cell survival of flavivirus infected cells (52). TMEM43 is a resident at the inner nuclear pore and, therefore, unlikely to directly influence HCV assembly. Therefore, we focused the validation of our screening approaches on RAD2B and its relative RAD23A. All three independent siRNAs and their pools consistently silenced the mRNA and protein levels of both RAD23A and B (Fig. 9B and C). Most, but not all, siRNAs also decreased HCV particle production (Fig. 9A). Additionally, we noted siRNA-dependent effects on cell viability and RNA replication (data not shown). Therefore, to avoid indirect effects caused by silencing of a host factor essential for cell survival, we overexpressed these proteins in Huh-7.5/FLuc cells, infected them with JcR2a, and monitored cell viability, HCV RNA replication, and infectious virus production by dual luciferase assays. Overexpression of RAD23A and B did not affect cell viability or HCV RNA replication, but it consistently increased production of infectious HCV progeny (Fig. 9D). Protein expression analysis via Western blot indicated a rather low, but visible, overexpression of Rad23B. In contrast, we detected a distinctly higher expression of Rad23A in cells transduced with lentiviral pseudo particles compared to the empty vector control. To ensure that we can replicate the effects of Rad23A and Rad23B knock-down on HCV JcR2a infection (Fig. 9A) with an untagged virus, we performed focus-forming unit (FFU) assays using the parental virus of JcR2a, Jc1, in Huh-7.5 cells (Fig. 9E). Supernatant of siRNA treated and infected cells was titrated on naϊve Huh-7.5 cells. Similar to our previous results, we could detect significantly reduced FFUs upon knock-down of Rad23A and Rad23B (Fig. 9F). Western blot analysis confirmed the knock-down of Rad23A and Rad23B on protein level (Fig. 9G).

## DISCUSSION

In this study, we surveyed protein-protein interactions of viral and host proteins involved in HCV particle production in human liver cells. We analyzed protein complexes during exponential virus production coordinated by two essential viral proteins (p7 and E2), a key cellular co-factor (ApoE), and a reference protein for viral replication (NS4B). Our quantitative proteome analysis revealed a broad range of novel protein-protein interactions between viral and host factors, and it showed common and distinct features of these complexes. Interactomes of HCV E2, p7, and ApoE were clearly distinct from those coordinated by NS4B, an integral component of HCV RNA replication organelles (40). The interactome of ApoE was completely different from the ones of p7, E2, and NS4B. In total, we identified five significant and specific ApoE interacting proteins. We noted 24 significant NS4B interacting proteins, of which only GTF3C3 also specifically interacted with HCV E2. These results suggest that HCV assembly and RNA replication occur in distinct ”factories,” which are separable from each other via affinity purification of E2 and NS4B. Collectively, these results support a model, where major constituents of HCV particles and their cellular interactors are deposited in separable cellular sites, which drive particle production and deliver components to assembling virions.

The majority of p7, E2, and ApoE likely is not directly associated with a virus particle. Following this assumption, the protein interactions captured with our analysis reflect the protein complexes within the cellular compartments from which these viral and host proteins are recruited into nascent particles. Recruitment of core protein from lipid droplets into motile puncta could be shown, which co-traffic with ApoE and with cellular adaptor proteins (AP) such as AP4, AP-1A/B, and AP-2 (53). Notably, p7, a transmembrane protein essential for HCV particle production (11, 17), interacts with NS2 (39, 54). Co-precipitation of adaptor proteins with p7 may be due to it interacting with NS2. Previously, others and we were unable to confirm a stable interaction between HCV E1-E2 and p7 (39, 54). Our proteomics analysis suggests a close interaction between p7, E1-E2 heterodimers, and NS2 because we noted a co-precipitation of both E2 and NS2 with HA-p7 (Fig. 2 and 3). Besides NS2, cellular PREB, STT3B, and SPCS2 also interacted with both p7 and E2. The observation that both p7 and E2 co-precipitated components of the signal peptidase complex (SEC11A for p7, SPCS2 for both p7 and E2, and SPCS3 as component of the E2 accessory interactome) is likely a reflection of the inefficient polyprotein processing at the E2-p7 site, which is due to structural determinants within p7 (51, 55). Prevention of polyprotein cleavage between E2 and p7 prevents infectious virus production. Furthermore, enforced separation of E2 from p7 via introduction of an internal ribosome entry site also decreased particle production (56). SPCS1 regulates E2-p7 cleavage and determines HCV assembly efficiency (51). Collectively, these data suggest that fine tuning of polyprotein processing between E2 and p7 may regulate E2 folding and trafficking of E2 protein complexes through the secretory pathway (possibly by recruitment of cellular adaptor proteins via p7-NS2) and budding of nascent virions into intracellular vesicles. Particle envelopment and budding may depend on release of p7 from E2. In support of this assumption, we recently provided evidence that p7 is essential for assembly stages prior to virus budding and capsid envelopment (13). Specific p7 interactors that we identified in the present study may reflect p7 interactions after E2-p7 cleavage and virus budding. Ineffective cleavage at the E2-p7 site may ensure recruitment of cellular adaptor proteins to nascent particles and thereby help routing of particles into transport vesicles. The complete processing at the E2-p7 sites may be important to sever nascent particles from membranes and permit particle egress. Such a regulatory mechanism may provide sufficient time for glycoprotein processing, folding, glycosylation, and oligomerization and serve as quality control for integration of fully folded and functional E1/E2 complexes into nascent virus particles. Accordingly, the primary proteome of HA-E2 reflects intense processing of HCV glycoproteins by ER-resident chaperones [calnexin (CANX), calmegin (CLGN)] and glycosylation [endoplasmic reticulum lectin 1 (ERLEC1), NGLY1].

We quantified changes to the HCV E2 proteome caused by disruption of distinct processes important for virus production. We employed virus mutants that we and others had characterized previously and which have defects in the uploading of core protein onto lipid droplets (core DP) (34–36), in the downloading of core from lipid droplets that is important for capsid envelopment (core C69-72A), and in the E1-E2 heterodimerization (E1 K179Q) (13, 38). Both core protein mutations profoundly influenced the E2 interactome (Fig. 5). Comparing these changes to the parental viral construct and between all mutants, we observed—among others—a significantly increased binding of oligosaccharyl transferases (STT3A and B), a chaperone (HSPA5), and RAD23A and B to the E2 protein in context of the core DP mutant (Fig. 7). These E2 interactomic alterations may be caused by the ablation of E1-E2 relocalization to assembly sites as recently described for other virus assembly-disrupting mutants (33). This relocalization was accompanied by wrapping of ER membrane carrying E1-E2 proteins around lipid droplets dependent on NS2, but not on core (33). This suggests that additional processes may be responsible for alteration of the E2 interactome in this viral mutant. Boulant et al. described a delayed cleavage of the core-E1 junction by the signal peptide peptidase in the JFH1-based viral core DP mutant (35). Thus, aberrant core-E1 processing may influence folding of E1 and in turn E2. Such difficulties in protein folding could enhance E2 binding to HSPA5. HSPA5, also known as BiP, is a chaperone responsible for correct folding of proteins within the ER and for transfer of misfolded proteins into the ERAD pathway for proteasomal degradation (57, 58). It was described as an interactor of hemagglutinin precursor HA0 aiding the correct folding of this viral protein for influenza virus (59). Moreover, an HSPA5 interaction with HCV E1 and E2 was noted before (60). HSPA5 may also identify proteins eligible for the ERAD pathway and channel them into this pathway for degradation (61, 62). Saeed et al. recently reported that HCV glycoproteins are targets of the ERAD pathway and that modulation of its components influences production of infectious particles (63).

Our interactome and STRING network analyses implicated additional cellular factors involved in the ERAD, ubiquitin, and proteasomal degradation pathways in the assembly and release of HCV (Fig. 6A). For instance, NGLY1 is one of the primary HA-E2 interactors and known to interact with the DNA repair protein complementing XP-C cells (XPC) factor Rad23B. ERLEC1 is also a member of the primary HA-E2 interactome and was suggested to be involved in the ERAD pathway by binding to misfolded proteins (64, 65). Since NGLY1 deglycosylates proteins, it might work upstream of Rad23 proteins to prepare misfolded viral proteins for proteasomal degradation. NGLY1 is also involved in major histocompatibility complex I depletion induced by human cytomegalovirus by deglycosylating the complex prior to proteasomal degradation (66, 67)

Rad23B plays a critical role in UV-induced nucleotide excision repair (NER) within the XPC. It contains an ubiquitin-like (UBL) domain, binding the 26S proteasome, and an ubiquitin-associated (UBA) domain, binding ubiquitin. Besides and in accordance with its function in NER, Rad23B is proposed to shuttle ubiquitinated proteins to the proteasome for degradation (46, 48) via interaction with a regulatory subunit of the 26S proteasome Rpn1 with its UBL domain (68). Consequently, Rad23B could serve as a shuttle service directing ubiquitinated HCV proteins toward degradation by the proteasome. Rad23A is a homolog of Rad23B, which we noticed in our STRING network analyses (Fig. 5A). Functionally, both proteins are redundant in terms of their role in stabilization and activation of the XPC complex (69, 70). Moreover, Rad23 proteins have been shown to be a part of the ERAD pathway in yeast cells (71) and to interact with N-glycanase (NGLY1; PNGase), which N-deglycosylates proteins prior to their proteasomal degradation (72). In a case-control study, it could be indicated that a single nucleotide polymorphism (SNP) rs1805329 (Ala249Val) in Rad23B was significantly associated with the development of hepatocellular carcinoma in HCV-infected patients (73). This SNP has also been connected to an increased risk of breast cancer (74).

Because HCV buds into ER-derived membranes, this cellular compartment is of major importance to the viral life cycle. Properly folded E1-E2 proteins are likely needed to drive particle assembly and budding. Misfolded and aggregated HCV E1-E2 proteins likely have to be deglycosylated and disposed of the ER to enable efficient virus assembly. Rad23B and its paralog Rad23A were most enriched with E2 in the context of the core DP mutant, which supports the notion that this mutation causes aberrant folding of E1-E2 complexes (Fig. 7A). Knock-down of RAD23A and RAD23B reduced HCV infectivity of tagged (Fig. 9A) as well as untagged virus (Fig. 9F). However, overexpression of these proteins slightly increased infectious virus production without affecting cell viability or replication (Fig. 9D). Our findings underpin the role of processing and folding as well as degradation of E1-E2 in controlling HCV assembly and infectious virus production. Moreover, it provides a landscape of protein-protein interactions, which define the protein composition of the subcellular compartments driving HCV particle production. Finally, our functional studies implicate Rad23A/B proteins as regulators of the ERAD pathways and in turn HCV particle production.

## MATERIALS AND METHODS

### Cell culture and virus stocks

Huh-7.5 or Huh-7.5/FLuc firefly luciferase reporter cells were used for infection assays described in this paper. Except for immunoprecipitations, Huh-7.5 cells were used. HEK293T cells (American Type Culture Collection, ATCC CRL-3612) were used for lentiviral particle production. Huh-7.5.1 (75) cells were used for virus production. All cell lines were cultured in Dulbecco’s modified Eagle’s medium (Gibco #41965039) with the addition of 10% fetal calf serum (Capricorn Scientific #FBS-11A), 100 U/mL of penicillin with 100 µg/mL of streptomycin (Gibco #15140122), non-essential amino acids (Gibco #11140050), and 2 mM L-glutamine (Gibco #25030024). To the medium of Huh-7.5/FLuc cells, 5 µg/mL blasticidin (Fisher bioreagents #BP2647-100) were added as a selection medium. Virus was produced on Huh-7.5.1 cells via electroporation of 5-µg RNA of the respective HCV construct into 6 × 10^6^ cells. Supernatant was collected after 48, 72, and 96 hours; pooled; filtered through 0.45-µm filters; aliquoted; and stored at −80°C.

### Plasmids and siRNAs

For virus production, the earlier described plasmids Jc1 pFK-JFH1/J6/XbaI/C-846 (76) and JcR2a (77) were used. Additionally, several mutants were included: Jc1 with double HA-tag at the N-terminus of p7, pFK-JFH1/J6/XbaI/C-846/HAHA-L-p7; Jc1 with the point mutation K179Q in E1, pFK-JFH1/J6/XbaI/C-846/E1 K179Q; Jc1 with double HA-epitope tagged p7 (N-terminal with G4SG linker) and with the point mutation K179Q in E1, pFK-JFH1/J6/XbaI/C-846/HAHA-L-p7/E1 K179Q; Jc1 with two distinct proline to alanine substitutions at aa residues 138 and 143, pFK-JFH1/J6/XbaI/C-846/Core P138A P143A; Jc1 with double HA-epitope tagged p7 (N-terminal with G4SG linker) and core protein with substitutions at aa residues 138 and 143, pFK-JFH1/J6/XbaI/C-846/HAHA-L-p7/Core P138A P143A; Jc1 with consecutive alanine residues at aa positions 69–72, pFK-JFH1/J6/XbaI/C-846/Core P138A P143A; and Jc1 with double HA-epitope tagged p7 (N-terminal with G4SG linker) and core protein with consecutive alanine residues at aa positions 69–72, pFK-JFH1/J6/XbaI/C-846/HAHA-L-p7/Core P138A P143A.

For overexpression of Rad23A and Rad23B, codon-modified genes (VectorBuilder) cloned into pLV[Exp]-Hygro-CMV> (VectorBuilder) were used. Sequence information is available upon request.

For RNAi experiments, Silencer Select siRNAs (Thermo Fisher Scientific) were used. For initial RNAi screens, the following siRNAs were used: AP2B1 (s36, s37, s38), ApoE (s1496, s194291, s1495), CANX (s2376, s2377, s2378), CLGN (s2882, s2883, s2884), CLIP1 (s12372, s12373, s12374), DCTN (s3973, s3974, s3975), DERL2 (s27226, s27227, s27228), ERLEC1 (s26042, s26043, s26044), FBXO2 (s25265, s25266, s25267), GNAB (s23247, s23248, s23249), GTF3C3 (s17832, s17833, s17828), HM13 (s37580, s224880, s195420), LARP4B (s23226, s23227, s23228), MESDC2 (s23223, s23224, s23225), NGLY1 (s31464, s31465, s31466), NIPSNAP (s16166, s16167, s16168), OS9 (s21564, s21565, s21566), PON2 (s10832, s10833, s10834), PREB (s19679, s19680, s19681), Rad23B (s11731, s11732, s11733), SEC11A (s23904, s23905, s23906), SEL1L (s12674, s12675, s12676), SH3BGRL2 (s38124, s38125, s38126), SPCS2 (s18918, s18919, 18920), STT3B (s47380, s47381, s47382), SYVN1 (s39019, s39020, 39021), TOMM40 (s20448, s20449, s20450), VDAC1 (s14768, s14769, s14770), VDAC2 (s14771, s14772, s14773), and VDAC3 (s14774, s14775, s230730). For experiments subsequent to the initial RNAi screens, individual or combinations of the following siRNAs were used: Rad23B-targeting siRNA (#1: s11731, #2: s11732, #3: s11733), Rad23A-targeting siRNA (#1: s11728, #2: s11729, #3: s11730), and the negative control no. 2 (#4390847) in a final concentration of 12 nM.

### Infection assays

For luminometer measurements, 1 × 10^5^ Huh-7.5/FLuc cells were seeded and infected 24 hours later with the reporter virus JcR2a, encoding Renilla luciferase. First-round infection was measured 48 hpi, and supernatants thereof were transferred to naïve Huh-7.5/FLuc cells for a second-round infection. First- (48 hpi) and second-round infections (72 hpi) were washed with phosphate buffered saline (PBS) and stopped via cell lysis by freezing samples in 200-µL H_2_O. Cell viability was measured via firefly luciferase counts, and whole replication cycle, via Renilla luciferase counts. First-round RLuc counts were normalized to first-round FLuc counts to account for cell viability effects, representing early replication cycle steps entry and replication. Second-round infection data were normalized to the previously normalized first-round infection, accounting for late replication cycle steps assembly and release of HCV virions.

### RNAi treatment and overexpression via lentiviral particles

For the first broad RNAi screen, Huh-7.5/FLuc cells (1 × 10^4^ cells/well) were seeded 4 hours prior to siRNA transfection using Lipofectamine RNAiMAX (#13778030, Thermo Scientific) according to the manufacturer’s manual. Conditions were tested in triplicates with 12.5 nM of three different siRNAs per target. Forty-eight hours post transfection, the cells were infected with the reporter virus JcR2a. Following medium change after 4 hours, the cells were kept for an additional 48 hours at 37°C before the supernatant was collected. Subsequently, the collected supernatant was used to infect naïve Huh-7.5/FLuc cells (1 × 10^4^ cells/well) 24 hours after seeding. After 72 hours, firefly luciferase signal was detected to quantify cell viability, while Renilla luciferase signal was used to measure HCV infectivity. Statistical analysis was performed in R using the RNAither package (50). Data were normalized to the negative controls, and Lowess normalization was employed to correct for cell count/viability differences. A z-score threshold of |2| and a *P*-value cutoff of <0.05 were used for hit calling. The z-score for each knock-down was calculated by subtracting the mean of the negative controls on the same plate and dividing by the standard deviation of the negative controls. Mean z-scores were calculated by averaging z-scores of all knock-downs targeting the same gene. Values *x* < 0 indicate a reduction, and *x* > 0, an increase of HCV replication or assembly and release compared to negative controls. On the y-axis, the log_10_ of the *P*-values of the scores are plotted.

For RNAi experiments with Rad23B and Rad23A in further experiments, the following siRNAs (see ”Plasmids and siRNAs” section above; final concentration: 12.5 nM) were transfected into 1 × 10^5^ Huh-7.5/FLuc cells via Lipofectamine RNAiMAX (Thermo Fisher). The following steps are described above.

Lentiviral particles were generated by transfecting 3.5 × 10^6^ HEK 293T cells with the vector pLV[Exp]-Hygro-CMV> comprising a hygromycine resistance gene as well as codon-optimized genes of Rad23B and Rad23A (VectorBuilder) as well as pCMA_ΔR8-74 and pVSVG using polyethylenimine. After 24 hours, 500 mM sodium butyrate was added. Forty-eight and 72 hours post transduction, the supernatant was pooled, filtered through 0.45-nm pore-size filters, and stored at −80°C. Target Huh-7.5/Fluc cells were transduced, and the medium was changed after 4 hours. Forty-eight hours post transduction, the cells were infected with JcR2a. The following steps were performed as described for the siRNA treatment above.

### Immunoblotting

SDS-PAGE followed by semi-dry immunoblotting was applied to transfer proteins. After 48 hours, the cells were lysed with RIPA buffer (1% Triton X-100, 2 mM EDTA, 0.1 M TRIS-HCl, 0.3 M NaCl in H_2_O) substituted with one Pierce Protease Inhibitor Mini Tablet per 10 mL (#A32953, Thermo Scientific). Primary antibodies for Rad23B (D4W7F, Cell Signaling rabbit mAb #13525,1:1000), Rad23A (D7U7Z, Cell Signaling, rabbit mAb #24555, 1:1,000), HAtag (clone 7, #A2095, Sigma Aldrich, mouse mAb, 1 µg/mL), and GAPDH (#G8795, Sigma Aldrich, mouse mAb, 1:5,000) were incubated overnight at 4°C. The secondary antibodies goat anti-mouse horseradish peroxidase (HRP) in 0.1 µg/µL concentration (A4416, Sigma Aldrich) and anti-rabbit-HRP in 1:20,000 concentration (AB_2307391, Jackson) were incubated for 1 hour at room temperature. For the Western blot in Fig. 9C, protein concentration was measured using the Qubit Protein Assay Kit (#Q33211, Invitrogen) according to the manufacturer’s instructions and measured with a Qubit Fluorometer 4 (Invitrogen). For Western blot in Fig. 9E and G, 8 × 10^4^ and 3 × 10^5^ cells, respectively, were loaded per lane. Samples were loaded onto selfcast gels (Fig. 9C) or NuPAGE 4% to 12% Bis-Tris 1.0-mm Mini Protein Gels, 15-well (#NP0323BOX, Invitrogen) (Fig. 9E and G). Proteins were transferred to Amersham Hybond P Membranes for western blotting, specificallyPVDF membranes (#GE10600023, Amersham). Protein signals were detected with the SuperSignal West Femto Maximum Sensitivity Substrate (Thermo Scientific) (Fig. 9C) or with ECL Western Blotting Detection Reagents (#RPN2106, Amersham) (Fig. 9E and G) and either the ChemoStar ECL imager (INTAS Science Imaging) (Fig. 9C) or the FUSION FX7 EDGE imager (Vilber) (Fig. 9E and G).

### Immunofluorescence

Immunofluorescence was applied to determine transfection efficiency. Respective cells (1 × 10^4^/well) were seeded on coverslips in 24-well plates, fixed with 3% paraformaldehyde after 24 hours, and permeabilized with 0.5% PBS-TritonX100. The primary antibody anti-NS5A clone 9E10 (kindly provided by Charles M. Rice) was incubated for 1 hour at room temperature in a concentration of 1 µg/µL. Following washing steps with PBS, the cells were incubated with the secondary antibody AlexaFlour−488 goat anti-mouse (#A32723, Thermo Scientific). Nuclei were stained with DAPI (1:10,000). Fluoromount (Southern Biotech) was used to fixate coverslips on glass slides and finally analyzed with the Olympus IX-81 confocal microscope and the FV1000 Viewer (Olympus). Samples were analyzed with ImageJ (Fig. 2). For the FFU assay, Huh-7.5 cells were stained with the anti-NS5A clone 9E10 antibody as described above and analyzed manually via the Olympus IX-81 confocal microscope.

### *In vitro* transcription (IVT) and transfection of viral RNA

Viral RNA was generated by IVTs. First, 10-µg plasmid DNA was linearized using MluI (New England Biolabs) and subsequently extracted via the QIAquick PCR Purification Kit (Qiagen) following the manufacturer’s recommendations. RNA elution was performed with 60-µL H_2_O. Then, linearized plasmid DNA was reverse transcribed [5× RRL, rNTPs, T7-polymerase (self-made), RNasin] for 2 hours at 37°C. After 2 hours, another 3-µL T7-polymerase was added followed by incubation for another 2 hours. Remaining DNA was digested with 7.5-µL DNase Q (Promega) for 30 minutes at 37°C. RNA was extracted (NucleoSpin RNA Clean-ip, Macherey Nagel) following the manufacturer’s instructions and stored at −80°C. Prior to immunoprecipitations, coupled to mass spectrometry experiments, viral RNA was transfected into Huh-7.5 cells. Electroporation was conducted with 5-µg viral RNA in a Gene pulser (Bio-Rad Laboratories) at 975 µF and 270 V. Huh-7.5 cells HA-ApoE overexpressing, with a shRNA-mediated knock-down of endogenous ApoE, and naϊve Huh-7.5 cells were electroporated with untagged Jc1 RNA.

### TCID_50_ assay

Huh-7.5 cells were seeded in a density of 1 × 10^4^ cells per well. The respective virus inoculum was titrated on the cells 24 hours post infection in a serial 1:10 dilution. The cells were fixed in ethanol 72 hours post infection as well as stained with the anti-NS5A clone 9E10 and anti-mouse-HRP antibodies. The HRP activity was visualized with a TCID_50_ detection substrate (76.92% acetatos, 0.23% carbazole, and 0.003% H_2_O_2_).

### FFU assay for the quantification of HCV infection in Rad23A and Rad23B knock-down cells

Huh 7.5 cells were seeded in 24-well plates in a density of 1 × 105 cells per well. After 24 hours, the cells were either transfected with siRNAs (Rad23A #3: s11730, Rad23B #3: s11733, negative control No. 2 #4390847, Thermo Fisher Scientific Silencer Select) as described above. The medium of all samples was changed 24 hours later. Forty-eight hours post transfection/transduction, the cells were infected with untagged Jc1 virus at an multiplicity of infection of 0.1 TCID_50_ per cell in a total volume of 370 µL. The medium was changed to 1 mL 4 hours post infection. The supernatant was collected 48 hours post infection and stored at −80°C. Thawed supernatant was titrated in 1:3 dilution steps 24 hours post seeding of Huh 7.5 cells in 96-well plates at a density of 1 × 104 cells per well. The top row was inoculated with 150 µL of the respective supernatants. Four hours post infection, 150-µL fresh medium was added to each well. Forty-eight hours post infection, the cells were fixed with methanol, and plates were stored at 20°C. For staining of NS5A, the plates were washed with PBS thrice and incubated with an anti-NS5A antibody [Cell Essentials, kindly provided by Charles Rice (78)] in PBS with 5% goat serum (Sigma Aldrich, #G9023, 1:2,000) for 1 hour at room temperature. Plates were again washed with PBS and incubated with a fluorescent goat anti-mouse antibody Alexa Fluor Plus 488 (#A32723, Invitrogen) in PBS with 5% goat serum for 1 hour at room temperature. Subsequently, the plates were again washed with PBS and stored at 4°C in the dark. FFUs were counted manually with an IX81 microscope (Olympus life science).

### Co-immunoprecipitation for mass spectrometric quantification

For both cell lysis and immunoprecipitation buffer, IP buffer was used (50 mM HEPES (pH 7.4), 150 mM NaCl, 10% (vol/vol) glycerol, 1% (vol/vol) NP-40, 1 mM CaCl_2_), which produced superior results compared to tested HC buffer [50 mM KOAc (pH 6.8), 2 mM MgCl_2_, 10% (vol/vol) glycerol, 1% (vol/vol) NP-40] in a prior test. Epitope-tagged complexes were pulled down via co-immunoprecipitation. Cell lysates were pre-cleared using control agarose resin (Thermo Scientific) for 1 hour. Subsequently, the lysate was incubated with an anti-HA-Agarose resin (#A2095, Sigma Aldrich) overnight at 4°C constantly rotating. For co-IPs of transfected hepatoma cells, lysates from a whole 10-cm cell culture dish were used. Antigens were eluted by heating up the resin with 4 M guanidine hydrochloride solution pH 8.5 (Sigma Aldrich) for 30 minutes at 900 rpm shaking. Co-immunoprecipitations of E2, NS4B, and ApoE were incubated at 70°C, whereas those for p7 were incubated at 37°C. The samples were stored at −80°C until mass spectrometric analysis. Since guanidine hydrochloride interfered with our control immunoblot analyses, we used 1-µL GlycoBlue coprecipitant (#AM9515, Invitrogen), 5 volumes of ethanol (99% analytical grade), and 0.04 volumes of 2.5 M sodium acetate (pH 5.0) for incubation at 4°C overnight for immunoblot samples.

### Mass spectrometry

Sample preparation, MS runs, and analyses of MS data were completed as previously described (50, 79). Protein eluates from co-immunoprecipitation were denatured with 6 M urea, reduced with 10 mM DTT, alkylated with 55 mM iodoacetamide, digested using LysC, diluted with H_2_O, and digested with trypsin. On the following day, the peptides were acidified with 0.1% TFA, desalted via reverse phased C18 tips, and separated via high-performance liquid chromatography. Subsequently, a nano electrospray ion source coupled to an orbitrap mass spectrometer (Q Exactive HF for the first data set, Q Exactive HF-X for the second data set, Thermo Scientific) was used to ionize peptides. Then, peptides were recorded via the Xcalibur software (Thermo Scientific). Mass spectrometric data were analyzed via the MaxQuant software [Max-Planck Institute of biochemistry, Martinsried, Germany (42)] additionally using the human Uniprot FASTA database (Version July 2015, 91647 entries), Andromeda search engine for a common contaminants databases (247 entries), and a full-length HCV Jc1 GT2a proteome. FDR was set to 1% on peptide and protein level. For a successful protein identification, at least one unique razor peptide with a length of minimum six amino acids per protein group was demanded. Quantifications were performed using the MaxLFQ label-free algorithms (80).

### Proteome and interactome analyses

Proteome data sets obtained from mass spectrometric analyses were analyzed utilizing Perseus software (Version 1.5.3.3, 1.5.5.5 and 1.6.1.1, Max-Planck Institute of biochemistry, Martinsried, Germany) (81). Protein hits were filtered for common contaminants, proteins only identified by one peptide or reverse hits. Then, label-free quantification (LFQ) intensities were logarithmized (log_2_), while missing values were imputed with values from a Gaussian distribution (width = 0.3, downshift = 1.7). Three to four biological replicates derived from hepatoma cells transfected with wt virus were grouped epitope-tagged viruses were compared to the untagged Jc1 wt control. IBAQ values were calculated by the MaxQuant software and indicate the relative amount of specific proteins. We used the MaxQuant software to calculate IBAQ values as a measure of protein abundance (42, 43). The IBAQ value was determined by dividing the total peptide precursor ion intensities by the number of theoretically observable peptides. Thereby, the IBAQ value allows an estimation of protein amounts (82). Statistical significance between each tested pair was tested via the non-parametric two-sample Welch’s *t*-test (FDR ≤0.05–0.1; S_0_ = 0–1, at least three valid values identified in the interactomes of the bait proteins; for p7, one valid value was accepted). For volcano plots, the logarithmic probability (P) was plotted against the LFQ intensity difference (log_2_). In the second data set, viral mutants were included, which arrest HCV assembly at certain steps. Filtering, logarithmization, and imputation were performed as described before. Two-sided Student *t*-test (FDR ≤0.05, S_0_ = 1, three valid values in the first group) was utilized to detect significant enriched hits for each viral variant. For detection of significant changes of protein-protein interactions of stage-dependent alterations in assembly, refined analyses were necessary. Total protein abundances of each samples were normalized to the respective bait protein. Groups of all independent biological replicates of each virus were formed. Multi-parametric two-sided *t*-test (FDR ≤0.05; S_0_ = 0; three valid values for the respective mutant) was applied together with the second analysis of variance (*P* ≤ 0.05) conducted for hits, which were found in the screen for factors showing stage-dependent regulation during assembly. Significant pairs were detected by applying post-hoc tests (FDR ≤0.05) and depicted by hierarchical clustering of z-scores.

### STRING analyses and KEGG pathways

To identify cellular factors interacting with our significantly enriched hits, which were pulled down with E2, p7, or ApoE HA-tagged bait proteins and subsequently enriched in mass spectrometry analysis, we performed STRING interaction network analysis (83–91). Significant hits from mass spectrometry analyses were entered into the freeware database. Showing a full network based on evidence collected from experiments and database search, with a confidence of 0.4 and no more than 50 interactors, we build a network surrounding our hit factors interacting with the bait proteins from our first mass spectrometry screen. Clustering was performed applying the Markov Cluster Algorithm with an inflation parameter of three. The network was edited in Adobe Illustrator. KEGG pathways and biological process (gene ontology) were exported from STRING and processed via Microsoft Excel 2016 and GraphPad Prism 9.

## Data Availability

The mass spectrometry proteomics data have been deposited to the ProteomeXchange Consortium via the PRIDE (92) partner repository with the dataset identifier PXD046941.
